# Pattern-Corrected Mitotic Activity Index (PMAI): A Novel Prognosticator of Oral Squamous Cell Carcinoma

**DOI:** 10.5146/tjpath.2019.01465

**Published:** 2020-01-15

**Authors:** Nunna Sai Chitra, Karen Boaz, Srikant N, Amitha J Lewis, Sneha K.S.

**Affiliations:** Department of Oral Pathology and Microbiology, Manipal College of Dental Sciences, Mangalore, Manipal Academy of Higher Education, Manipal, Karnataka, India

**Keywords:** Oral cancer, Mitotic index, Prognosis, Survival, Squamous cell carcinoma

## Abstract

***Objective:*** The main aim was to assess the efficiency of the Mitotic Activity Index (MAI) and a novel index devised by us, the Pattern-Corrected Mitotic Activity Index (PMAI) in prognostication of Oral Squamous Cell Carcinoma in terms of lymph node involvement, margin, recurrence and survival status.

***Material and Method:*** The study group consisted of 60 cases of histologically-proven Oral Squamous Cell Carcinoma with known status of prognostic indicators. Hematoxylin and eosin stained sections of the tumor proper were utilized for assessment of mitotic activity and pattern of invasion. The Mitotic Activity Index and Pattern-Corrected Mitotic Activity Index were then calculated and correlated with the prognosticators.

***Results:*** Mitotic Activity Index was higher in patients who had better survival and low recurrence rates. Pattern-Corrected Mitotic Activity Index showed the greatest percentage increase in relation to lymph node involvement as compared to the other indices. Kaplan Meier survival analysis showed that a higher Pattern-Corrected Mitotic Activity Index (>1.45) was associated with poorer survival (37.19 months).

***Conclusion:*** Lack of significant association of the Mitotic Activity Index in relation to prognosticators could be attributed to a tumor having a migratory phenotype rather than a proliferative phenotype as seen in late-stage tumors. Late-stage tumors have more of a poorer pattern of invasion which is reflected best by Pattern-Corrected Mitotic Activity Index by correlating with poorer survival and lymph node involvement.

## INTRODUCTION

Head and neck malignancies constitute the majority of cancers among the Indian population. The prevalence of Oral Squamous Cell Carcinoma (OSCC) is increasing over the last decades, with a high incidence of associated morbidity and mortality. Despite enormous advancements in the field of diagnostics and therapeutics, the overall survival rate in most countries has not shown significant improvement. The prognosis remains quite unclear as the involvement of lymph nodes and surgical margins by tumor directly impacts on recurrence and survival.

TNM staging and histological grading systems have been used commonly to prognosticate OSCC (1). Bryne M. et al. evaluated five histological features namely degree of keratinization, nuclear pleomorphism, lymphoplasmacytic infiltrate, the pattern of invasion and mitotic activity to grade the carcinoma at the Invasive Tumor Front (ITF), which is popularly regarded as a reliable prognosticator of OSCC. Studies later have assessed individual histological parameters to determine their efficiency in prognostication of OSCC. The assessment of cellular proliferation is one such parameter used not only for primary diagnostic purposes but also as a guide to prognosis. Several sophisticated techniques are available for assessing proliferative activity of tumors; however, the oldest, easiest, fastest and cheapest way of assessing proliferation is by counting the number of mitoses in tissue sections. Various histological grading systems have incorporated mitotic activity and pattern of invasion in their assessment with the earliest grading systems having a subjective evaluation of mitosis whereas, the later systems having an objective way of assessment of mitosis. The assessment of mitotic activity is one of the cornerstones for the diagnosis and grading of OSCC, soft tissue sarcomas and other malignancies. Mitotic activity index (MAI) is the oldest way of measuring proliferative activity. It is cost effective as mitoses are counted in routinely fixed and processed hematoxylin and eosin (H&E) sections and if performed carefully can give very useful information (2).

The pattern of invasion at the ITF in the form of individual cells or strands is known to be an adverse prognosticator of OSCC. The loss of adhesive proteins results in a dissociative pattern of invasion visualized histologically as individual cells and strands. We hypothesized that the tumors showing the worse pattern of invasion (like permeating as individual cells and thin strands) having concomitantly higher mitotic activity would have a poorer prognosis. Thus, we hereby propose a novel mitotic index that combines the two parameters as the Pattern-Corrected Mitotic Activity Index (PMAI) (2).

Therefore, this study intended to objectively and comparatively assess mitotic activity along with a new proposed Pattern-Corrected Mitotic Activity Index, in an attempt to predict the prognosis of OSCC.

## MATERIALS and METHODS

### Sample Selection

Formalin-fixed, paraffin-embedded tissue blocks of 60 cases of OSCC were retrieved from the archives of the Department of Oral Pathology, MCODS, Mangalore. Relevant patient data (TNM staging, recurrence, and survival) was accessed from the medical records file of the patient. The histopathological diagnosis of margins and the lymph node status were assessed by retrieving the slides and histopathological reports. Patients who underwent curative excision as the primary mode of treatment were included in our study. The study commenced after approval from the Institutional Ethics committee

Sections of 4μm thickness were taken from the representative paraffin-embedded tissue blocks and were subjected to routine H&E staining. The archived slides were analyzed in correlation with the grossing sheet, to identify the invasive tumor front of the OSCC. Brynes’s grading system was employed at the Invasive Tumor Front and the number of mitotic figures and the pattern of invasion were assessed for each case. The tumor was assessed in 10 HPF. A 100-point square grid reticule (Olympus, 190cm 10x10 square) was placed in the eye-piece of the Olympus CH20i microscope. The tumor cells were assessed at the invasive tumor front with the grid superimposed on the field of view. Using the grid as a reference, the number of mitotic figures were counted in the given fields.

The number of mitotic figures was assessed in the corresponding fields as per the criteria proposed by van Diest et al. (3). The cell under consideration should not have a nuclear membrane with the nuclear material having clear ‘hairy’ extensions (condensed chromosomes) present either clotted together (beginning metaphase), in a plane (metaphase/anaphase) or in separate clots (telophase). Structures and figures not satisfactorily meeting the above-mentioned criteria were excluded from the assessment.

The pattern of invasion score was assessed in each field using Bryne’s Grading criteria where a score of 1 was given for pushing, well-delineated infiltrating borders, score 2 for infiltrating, solid cords, bands, and strands, score 3 for smaller groups of cells having >15 cells and score 4 for tumor showing marked widespread cellular dissociation in small groups (n<15) or in individual cells (2).

Following the counting of mitotic figures in 10 high power fields, and pattern of invasion the following mitotic indices were employed. The PMAI has been newly derived by us as a potential prognosticator as it combines the pattern of invasion and mitotic activity of the tumor.

• MAI (Mitotic Activity Index) was defined as the number of mitotic figures in 10 HPFs.

• PMAI (Pattern-corrected Mitotic Activity Index) was calculated as the average of the product of the mitotic figures and pattern score in ten fields.

### Statistical Analysis

Demographic data for 60 cases were summarized using descriptive analysis. Prognostication of OSCC was done using the parameters of pathological lymph node involvement, margin status, recurrence and survival of the patient. The association of prognosticators with TNM staging was done using the chi-square test. MAI and PMAI were compared between the better and the worse prognosis of the above parameters using independent student t-test. The mitotic indices were compared with the four stages of the TNM staging system using the Kruskal Wallis test. ROC curve analysis was performed to assess the area under the curve and predictive efficiency (for a given cut-off) of each mitotic index in relation to the prognosticators. The cut-off for each mitotic index was arrived at using the coordinates having the highest sensitivity and specificity for the event of lymph node involvement. Using the cut-offs so derived, the sensitivity, specificity, positive predictive value, negative predictive value and diagnostic accuracy was calculated for each prognostic parameter.

Kaplan Meier Survival analysis was performed for 60 patients and a comparison of the mean age of survival was performed at the derived cut-off of each mitotic index using the Log-Rank test.

## RESULTS

In a cohort of 60 cases of OSCC, we have observed the predominance of males (81.7%) with a varying age group of 34 to 80 years and a mean age of 57.5±11.13 years. Most of the cohorts were placed in TNM Stages IV and III (58.3% and 26.7% respectively). Brynes’ histological grading system was used to evaluate the predominant pattern of invasion as well as the worst pattern of invasion. Lymph nodes were free of tumor in 73.3% of the cases and surgically resected margins were found to be involved in two-thirds of the cases (65.0%). Also, 55.0% of OSCC patients showed no recurrence and the survival rate of the cohort was 75.0% (Table 1).

**Table 1:** The demographic features of the patients.

		**Number**	**%**
Age (34- 80 years) (Mean ± SD: 57.5 ± 11.13)	<40 years (36.750 ± 2.754)	4	6.7
	41-50 years (46.000 ± 2.972)	13	21.7
	51-60 years (55.412 ± 3.501)	17	28.3
	61-70 years (65.158 ± 2.853)	19	31.7
	>70 years (75.000 ± 3.266)	7	11.7
Gender (Mean ± SD)	Female (60.182 ± 8.750)	11	18.3
	Male (56.898 ± 11.589)	49	81.7
Site	Alveolus	16	26.7
	Buccal mucosa	18	30.0
	Floor of the mouth	5	8.3
	Maxillary alveolus, palate	5	8.3
	Tongue	16	26.7
Tumor stage	T1	10	16.7
	T2	30	50.0
	T3	10	16.7
	T4	10	16.7
Lymph node stage	N0	6	10.0
	N1	22	36.7
	N2a	7	11.7
	N2b	14	23.3
	N2c	11	18.3
Metastasis	M0	33	55.0
	Mx	27	45.0
TNM stage	Stage I	5	8.3
	Stage II	4	6.7
	Stage III	16	26.7
	Stage IV	35	58.3
Lymph node status (Resected nodes)	Free	44	73.3
	Involved	16	26.7
Margin status	Free	21	35.0
	Involved	39	65.0
Recurrence	No recurrence	33	55.0
	Recurrence	27	45.0
Survival status	Alive	45	75.0
	Dead	15	25.0
Bryne’s grade with predominant pattern of invasion	Well-differentiated	12	20.0
	Moderately-differentiated	37	61.7
	Poorly-differentiated	11	18.3
Bryne’s grade with worst pattern of invasion	Well-differentiated	10	16.7
	Moderately-differentiated	36	60.0
	Poorly-differentiated	14	23.3

Mitotic indices like Mitotic Activity Index (MAI) and Pattern-corrected Mitotic Activity Index (PMAI) were compared with the prognostic parameters like lymph node involvement, margin recurrence, and survival. Pattern-corrected Mitotic Activity Index and Mitotic Activity Index were higher in cases with lymph node involvement showing 49.01% and 20.38% change respectively. In the cases showing the recurrence of tumor, poor survival and positive surgical margins it was noted that the mitotic indices were lower (Table 2).

**Table 2:** Comparison of The MAI and PMAI between the prognostıc factors using independent student T test.

** **	** **	**Status**	**N**	**Mean**	**Std. Deviation**	**Percentage change**	**t**	**P value**
Lymph node	MAI	Free	44	8	7.87	-20.38	-0.738	0.464
		Involved	16	9.63	6.52			
	PMAI	Free	44	2.02	2.05	-49.01	-1.598	0.115
		Involved	16	3.01	2.3			
Margin status	MAI	Free	21	9.62	7.61	19.02	0.895	0.374
		Involved	39	7.79	7.49			
	PMAI	Free	21	2.68	2.37	22.76	1.046	0.3
		Involved	39	2.07	2.02			
Recurrence	MAI	No recurrence	33	8.61	7.69	4.53	0.195	0.846
		Recurrence	27	8.22	7.44			
	PMAI	No recurrence	33	2.35	2.37	6.38	0.265	0.792
		Recurrence	27	2.2	1.88			
Survival	MAI	Alive	45	9	7.3	25.22	1.012	0.316
		Dead	15	6.73	8.15			
	PMAI	Alive	45	2.44	2.07	25.82	0.99	0.326
		Dead	15	1.81	2.37			

**MAI:** Mitotic Activity Index, **PMAI:** Pattern-Corrected Mitotic Activity Index.

**Figure 1:**
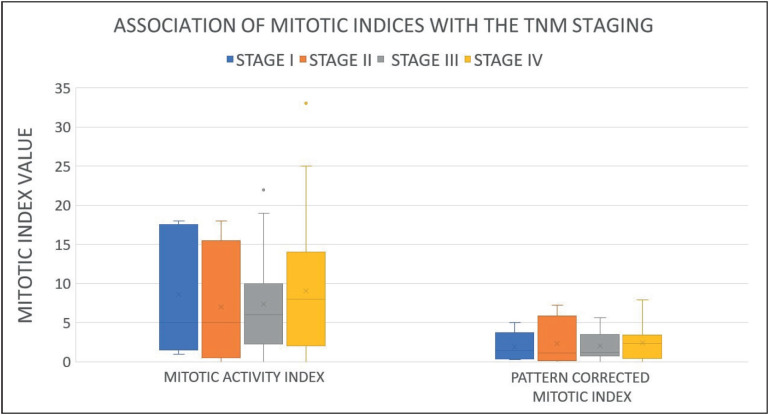
Median scores of Mitotic indices in relation to the TNM staging system (Box and Whisker Plot).

MAI and PMAI were compared with TNM staging using the Kruskal Wallis test and were seen to show the highest median scores in stage IV tumors. The Mitotic Activity Index showed a gradual increase from stage I through stage IV and PMAI showed higher scores in stage I with a lower score in stage II which increased in stage III and stage IV (Figure 1).

Receiver Operator Curve (ROC) Characteristics was performed for each of the prognosticators with the mitotic activity indices. The area under the curve was more than 0.5 only in the prediction of lymph node involvement, indicating that mitotic activity was correlating with the lymph node involvement and not with margin, recurrence or survival in our cases. Among the mitotic indices, the higher area under the curve was shown by PMAI (0.663) compared to MAI (0.608). Using the coordinates of the curve based on the ROC curve for prediction of recurrence, we arrived at an optimum cut-off of 7.5 for MAI and 1.45 for PMAI showing similar sensitivity/specificity of 62.50% and 56.80%.

**Figure 2:**
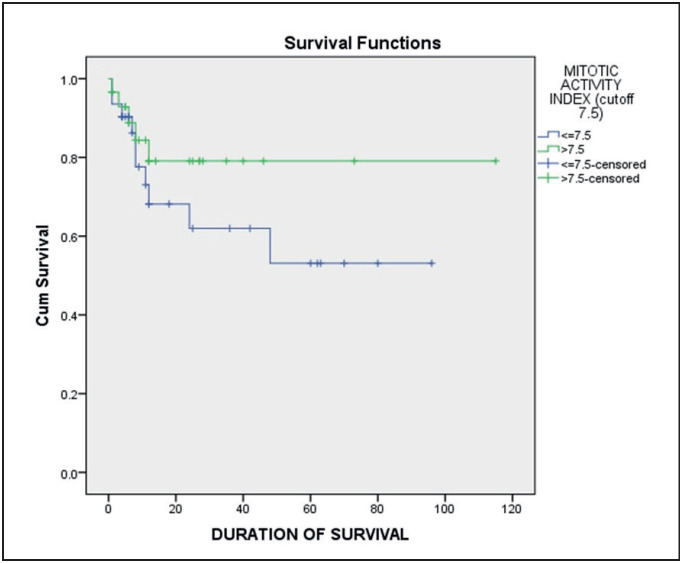
Kaplan Meier survival plot for MAI with a cut-off of 7.5.

**Figure 3:**
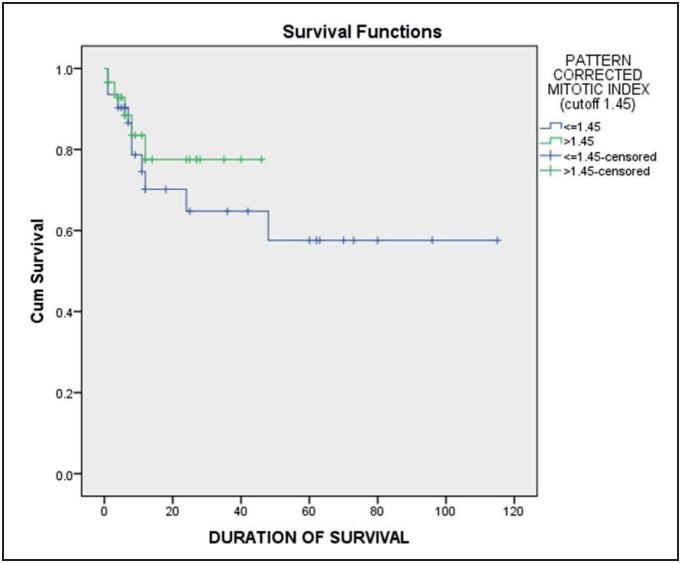
Kaplan Meier survival plot for PMAI with a cut-off of 1.45.

Kaplan Meier survival analysis showed that MAI had better survival with higher values of the index. Patients with MAI >7.5 had a mean survival rate of 92.33 months compared to 58.98 months in patients with scores <7.5. However, the Pattern-Corrected Mitotic Activity Index had an inverse association. Patients with PMAI >1.45 showed poorer survival of 37.19 months as compared to the ones having <=1.45 PMAI with a mean survival of 73.02 months (Table 3) (Figure 2 Figure 3).

**Table 3:** Kaplan Meier Survival Analysis.

**Parameter**	**Cut-off**	**N**	**Mean estimation of survival**	**Standard error**	**95% Confidence Interval**		**Chi Square**	**P value**
					**Lower**	**Upper**		
MAI	<=7.5	31	58.98	9.08	41.19	76.78	1.208	0.272
	>7.5	29	92.33	9.16	74.37	110.29		
PMAI	<=1.45	31	73.02	10.52	52.41	96.63	0.529	0.467
	>1.45	29	37.19	3.53	30.27	44.11		

**MAI:** Mitotic Activity Index, **PMAI:** Pattern-Corrected Mitotic Activity Index.

Binary logistic regression analysis shows that an involved margin status has the highest odds of death with odds ratio of 5.961. This is followed by a pattern-corrected mitotic index which shows odds of 3.331 toward death when the PMAI is <=1.45. Buccal mucosa involvement showed higher odds of 2.009 compared to alveolus involvement in event of death (Table 4).

**Table 4:** Binary logistic regression analysis (enter method) for assessment of the odds of survival.

		**N (60)**	**B**	**S.E.**	**Wald**	**P value**	**Odds ratio**	**95% C I .for odds ratio**	
								**Lower**	**Upper**
**Variables**	Age		-.027	.035	.604	.437	.973	.909	1.042
	Gender (Female)#	11	-1.058	1.001	1.117	.291	.347	.049	2.470
	Alveolus (Ref)	16			2.603	.626			
	Buccal mucosa	18	.698	.878	.632	.427	2.009	.360	11.226
	Floor of the mouth	5	.004	1.482	.000	.998	1.004	.055	18.325
	Maxillary alveolus/Palate	5	-.212	1.629	.017	.897	.809	.033	19.702
	Tongue	16	-.927	1.015	.835	.361	.396	.054	2.892
	TNM stage I (Ref)	5			2.912	.405			
	TNM stage II	4	-20.153	19604.622	.000	.999	.000	.000	.
	TNM stage III	16	-1.891	1.601	1.396	.237	.151	.007	3.478
	TNM stage IV	35	-.396	1.452	.075	.785	.673	.039	11.583
	Recurrence (Positive)	27	.137	.705	.038	.846	1.147	.288	4.563
	Margin Status (involved)	39	1.785	.981	3.311	.069	5.961	.871	40.766
	Lymph node status (Involved)	16	-.011	.820	.000	.989	.989	.198	4.934
	PMAI (<=1.45)##		1.203	.778	2.389	.122	3.331	.724	15.318
	Constant		-.620	2.723	.052	.820	.538		

#: Reference male, ##: Reference >1.45, **PMAI:** Pattern-Corrected Mitotic Activity Index.

## DISCUSSION

Proliferation is one of the fundamental processes in neoplasia as it plays a critical role in the growth and sustenance of tumor. Tumor volume maintenance needs multiplication of cells that in turn requires the process of mitosis. Proliferation markers have been utilized by many researchers in a bid to predict prognosis. The simplest method of assessing the proliferation is by evaluation of the number of mitosis which can be readily identified by routine H&E staining. The clinical TNM staging and histological grading (Bryne’s Invasive tumor front grading system) have been the standards in the prediction of prognosis. One of the components of TNM staging is the size of the tumor which characterizes the tumor load or the quantity of the tumor along with the nodal status and metastatic character (4).

Bryne’s grading system has included mitotic figures along with other histological characteristics like maturation, pleomorphism, host response and pattern of invasion and the grading system has yielded excellent correlation with prognosis. From amongst the other parameters of Bryne’s grading system, the ‘pattern of invasion’ may be regarded as an indicator of the migratory potential of the tumor. Fundamental to neoplasia is the requirement of cells to multiply and go forth to spread and metastasize (5).

Studies have shown that the pattern of invasion and mitotic activity are the two most important prognosticators (6,7). We developed the Pattern-corrected Mitotic Activity Index (PMAI) to validate our assumption that tumors with high proliferation and greater migratory potential would have a poorer prognosis. The novel ‘Pattern-corrected Mitotic Activity Index’ (PMAI) quantifies the combination of the mitotic activity and the pattern of tumor invasion at the invasive tumor front.

In our study, MAI was correlated with prognostic parameters and was seen to be higher in patients who had better survival rates and low recurrence rates. These results were similar to those reported by Davies et al. who immunohistochemically assessed the proliferation rate of oral squamous cell carcinoma using Ki-67 and found a higher recurrence rate to be associated with lower proliferation index (8). Veronica A et al. have also reported higher survival rates correlating with higher rates of AgNOR’s and Ki67, in oral squamous cell carcinoma of a cohort in Uruguay (5).

Survival in patients with OSCC depends on multiple factors. Dissanayaka et al. assessed a cohort of 193 patients with OSCC and found a significant association between the pattern of invasion of tumor and nodal metastases. They reported that patients with tumors invading in individual cells and thin strands showed a higher tendency for metastasis with poorer survival (mean of 2.7 years) as compared to the patients with tumor invasion islands or as broad pushing margins (mean survival of 3.7 years). Thus a tumor having a low mitotic activity but a worse pattern of invasion would correlate with poorer survival owing to the higher metastatic potential of the dissemination of tumor cells. This is demonstrated by the PMAI index efficiently (9). The PMAI index has the ability to quantify the two significant prognosticators of OSCC, namely, proliferation (mitosis) and potentially migratory component (pattern of invasion) in a unified single index. This novel index (PMAI) proved to be an efficient prognosticator in our cohort as it showed a positive correlation with mortality. Patients with a higher PMAI score of >1.45 had a lower mean survival rate of 37.19 months as compared to the ones with PMAI<=1.45 who survived for nearly twice as long (73.02 months) (Table 3).

Lymph node (N) status is known to be the most important clinical parameter determining survival. PMAI showed a greater percentage increase of 49.01% in relation to lymph node involvement as compared to the Mitotic Activity Index (20.38%).

Our lack of observed significant correlation between nodal status and recurrence, margin and survival could be attributed to a bias in the cases in our cohort wherein most patients were in late-stage tumors constituting, TNM stages III and IV owing to the nodal status being either N1 or N2 in 54/60 cases. In late-stage tumors the neoplastic cells are more likely to have acquired a mesenchymal phenotype that supports migration. In what could be simplistically described as a ‘change of priorities’, the tumor that previously focused on volume has now changed track towards metastasis and to achieve this change in focus, the resultant epithelial-mesenchymal transformation that involves reorganization of cytoskeleton has since rendered the cell incompatible for significant proliferation to occur (5).

This phenomenon is supported by studies by Bettendorf and Herrmann who indicated that the tumor growth is exponential at its onset whereas in later stages the cell cycle slows down. This was assessed by immunohistochemical markers like Ki67, PCNA, and P34, which indicate the increasing DNA synthetic activity in the cell, which is higher in the early stage tumors (10). Freudlsperger et al. found that Ki-67 labeled proliferation positively correlated with increasing frequency of loco-regional recurrence in stage I tumors while tumors in Stages III and IV in their cohort showed a lack of correlation of proliferation with survival or loco-regional recurrence. This is in agreement with our findings as most of the OSCC cases in our cohort were in stages III and IV suggesting that the tumor has ceased to be in active proliferation mode and is progressing towards acquiring phenotypical changes in preparation for epithelial-mesenchymal transformation and eventual migration (11).

It is understood that the worsening TNM stage increases the potential for metastasis as is evident by the shift in the pattern of the tumor phenotype. After a threshold in tumor size is achieved, the tumor environment is no longer conducive for proliferation. This initiates the phenotypic switching indicated by a reduction in the proliferation markers and an increase in factors associated with the migration of tumor cells. Thus, in late-stage tumors, one can expect lower proliferation rates as we did find upon correlation with mitotic indices.

Lack of a significant correlation of the mitotic indices with prognosis could also be attributed to other factors governing tumor biology.

It may be the quality and not merely the number of mitoses that may correlate with the prognosis. The presence of tripolar, tetrapolar, and atypical anaphase mitotic figures indicate the presence of genetic aberrations and correlate with aneuploidy.

The presence of an increased number of mitotic figures may just be related to the increased response to growth factors rather than conferring aggression on the tumor.

As the number of cell divisions increase, tumor cells progressively acquire and accumulate genetic aberrations that may propel the tumor cells towards apoptosis or migration. As explained earlier, early-stage tumors are characterized by mitotic activity and tumor cells in the later stages are more inclined to migrate and metastasize rather than divide. Thus, mitotically active/dividing cells will correlate with the size of the tumor but not the metastasis, margin, recurrence or survival status of the individual.

In conclusion, the efficacy of mitotic indices that assess mitotic activity alone or in conjunction with tumor volume is questionable in predicting prognosis in late-stage tumors. Late-stage tumors acquire a migratory phenotype that thrives less on proliferation through mitosis and more on the ability to metastasize. Therefore, the PMAI offers the prospect of being a better predictor of survival as it incorporates assessment of mitosis along with the pattern of tumor cell invasion.

## Conflict of Interest

The authors declare no conflict of interest.
